# A Novel Murine Model of a High Dose Brachytherapy-Induced Actinic Proctitis

**DOI:** 10.3389/fonc.2022.802621

**Published:** 2022-02-23

**Authors:** Carlos Heli Bezerra Leite, Carlos Diego Holanda Lopes, Caio Abner Vitorino Gonçalves Leite, Dulce Andrade Terceiro, Gabriel Silva Lima, Jéssica Andrade Freitas, Fernando Queiroz Cunha, Paulo Roberto Carvalho Almeida, Deysi Viviana Tenazoa Wong, Roberto César Pereira Lima-Júnior

**Affiliations:** ^1^Radiation Oncology Service, Haroldo Juaçaba Hospital, Cancer Institute of Ceara (ICC), Fortaleza, Brazil; ^2^Drug Research and Development Center, Department of Physiology and Pharmacology, Faculty of Medicine, Federal University of Ceara, Fortaleza, Brazil; ^3^Department of Pharmacology, School of Medicine of Ribeirão Preto, University of São Paulo, Ribeirão Preto, Brazil; ^4^Department of Pathology and Forensic Medicine, Faculty of Medicine, Federal University of Ceara, Fortaleza, Brazil

**Keywords:** brachytherapy, rectum, inflammation, animal model, actinic proctitis

## Abstract

**Background:**

Radiation proctitis affects 1-20% of cancer patients undergoing radiation exposure due to pelvic malignancies, including prostate, gynecological and rectum cancers. The patients manifest rectal discomfort, pain, discharge, and bleeding. Notably, the efficacy of prophylactic measures remains controversial due to the lack of adequate animal models that mimic this condition.

**Objective:**

The present study then aimed to develop a murine model of high-dose-rate (HDR) brachytherapy-induced proctitis.

**Material/Methods:**

C57BL/6 male mice were subjected to HDR (radiation source: iridium-192 [Ir-192]) through a cylindrical propylene tube inserted 2 cm far from the anal verge into the rectum. The animals received radiation doses once a day for three consecutive days (fractions of 9.5 Grays [Gy]), 3.0 mm far from the applicator surface. The sham group received only the applicator with no radiation source. The survival rate was recorded, and a colonoscopy was performed to confirm the tissue lesion development. Following euthanasia, samples of the rectum were collected for histopathology, cytokines dosage (IL-6 and KC), and immunohistochemical analysis (TNF-α and COX-2).

**Results:**

HDR significantly reduced animals’ survival ten days post first radiation exposure (14% survival vs. 100% in the non-irradiated group). Day seven was then used for further investigation. Mice exposed to radiation presented with rectum injury confirmed by colonoscopy and histopathology (*P* < 0.05 vs. the control group). The tissue damage was accompanied by an inflammatory response, marked by increased KC and IL-6 tissue levels, and immunostaining for TNF-α and COX-2 (*P* < 0.05 vs. control group).

**Conclusions:**

We established a novel animal model of actinic proctitis induced by HDR brachytherapy, marked by inflammatory damage and low animal mortality.

## Introduction

Radiation proctitis is a side effect that affects cancer patients undergoing radiation exposure due to pelvic malignancies (1). Despite the progressive improvement in radiation techniques, the incidence of such toxicity remains observed since radiotherapy is used in association with other anticancer therapies for many types of cancer (2). Notably, radiation proctitis can be far more detrimental than the neoplasia for which the treatment was indicated, as described in some cases of low-risk prostate cancer (3).

Patients who develop radiation proctitis experience rectal discomfort, pain, and discharge or bleeding, with the consequent need for endoscopic interventions (1). The efficacy of prophylactic measures, including daily intravenous use of amifostine prior to therapy (4) or sucralfate administered by topical or oral routes (5), remains controversial (1). Clinical management of radiation proctitis comprises amifostine, mesalazine, sucralfate, formalin local application, electrocoagulation, Nd-YAG laser, and hyperbaric oxygen therapy. However, all these therapeutic options present poor outcomes (1, 6). More invasive and aggressive therapies, including rectal resection (3), are sometimes requested, increasing healthcare costs and reducing patients’ quality of life (3). Therefore, more effective therapeutic approaches are an unmet need. Understanding the pathophysiology and molecular mechanisms of the underlying inflammatory response could then contribute to identifying potential therapeutic targets and open perspectives for explaining different disease patterns even in patients who have been treated with the same radiation dose.

To investigate the efficacy of potential drugs for preventing or treating radiation proctitis in pre-clinical studies, a well-established animal model in which rectal damage accompanied with minimal mortality is highly demanded. Ashcraft and colleagues reported a murine model of chronic radiation-induced proctitis using an X-RAD 225-Cx (Precision X-Ray) small animal irradiator, multiple plan configurations, and delivering a 15 Gy 3D conformal treatment plan from a scanned reference mouse. Their irradiation resulted in 40% mortality at 250 days and no acute mortality (7). Histopathological analysis showed fibrosis of the irradiated colon and increased mucous production.

Contrasting with the X-ray-based model by Ashcraft and colleagues, high-dose-rate (HDR) iridium brachytherapy is widely used and commonly available in radiation oncology centers and seems to be the ideal radiotherapy modality to induce radiation damage in mice due to its feasibility, the small dimension of the radioisotope source and compatibility with the cylindrical rectal anatomy. These characteristics make fractionated radiotherapy protocols easier to be tested. Brachytherapy can also limit the dose to the rectum, avoiding unnecessary irradiation and damage to other structures beyond the external beam. There is currently only one HDR brachytherapy-based proctitis mouse model but limited reproducibility due to the extensive radiation schedules used (8). That study only evaluated the late radiation damage failing to investigate the time course of the disease. Such knowledge would ideally contribute to identifying more insidious targetable inflammatory mediators. In addition, none of the previous models has used colonoscopy to evaluate radiation damage.

Therefore, the purpose of this study was to delineate a novel animal model of actinic proctitis using HDR brachytherapy in mice and to evaluate the expression of inflammatory markers. The present study used a dosing schedule of three fractions of 9.5 Gy, representing a biologically effective dose (BED) of 96.2 Gy4. It was based on dosing schedules previously investigated in rodent models of proctitis (7, 8).

## Materials and Methods

### Animals

C57BL/6 mice (20-24 g, 6-8 weeks old) were obtained from the animal facility of the Drug Research and Development Center, Federal University of Ceará (Fortaleza, Brazil). The Ethics Committee on Animal Use approved the study (approval number 50/13). The animals were kept in propylene cages (6 mice/cage) with environmental enrichment and a temperature-controlled room (23 ± 1°C) with 50-60% relative humidity. The mice were submitted to a 12h/12h light-dark cycle with free access to food (Nuvilab CR1, São Paulo, Brazil) and water. Bedding consisting of gamma-ray irradiated pine wood shavings (Suzano, São Paulo, Brazil) was changed twice a week. We allocated the animals into equal-sized groups (6 animals per group). The animals received ketamine (80 mg/kg, i.m) and xylazine (16 mg/kg, i.m.) for anesthesia, followed by cervical dislocation for euthanasia.

### Sample Size

The sample size in this study was calculated based on a pilot study to determine the capacity of irradiation to induce a significant cytokine-driven inflammatory response. In that step, we measured the levels of IL-6 and KC in colon samples of irradiated and non-irradiated mice. For both cytokines, the minimum required number of mice per group was set as five. The calculated sample size was then added by one mouse, considering the potential loss of animals during the experiments. The formula for calculating sample size when comparing two means, with an alpha error of 5% (Z_α/2_ = 1.96) and a beta error of 80%, is as follows: $n=(s_{1^{2}}+s_{2^{2}})*{\left(Z_{\alpha /2}+Z_{\beta }\right)}^{2}/{\left({\bar{x}}_{1}-{\bar{x}}_{2}\right)}^{2}$ , where *n* = number of animals required in each of the two groups; $s_{1^{2}}+s_{2^{2}}$ = mean estimated variance of the groups to be compared; ${\bar{x}}_{1},{\bar{x}}_{2}$ = the means of the groups to be compared (9). A total of 118 animals were used in this study (**Supplementary Data Table 1**).

### Induction of Experimental High-Dose Radiation Proctitis

**Figure 1:**
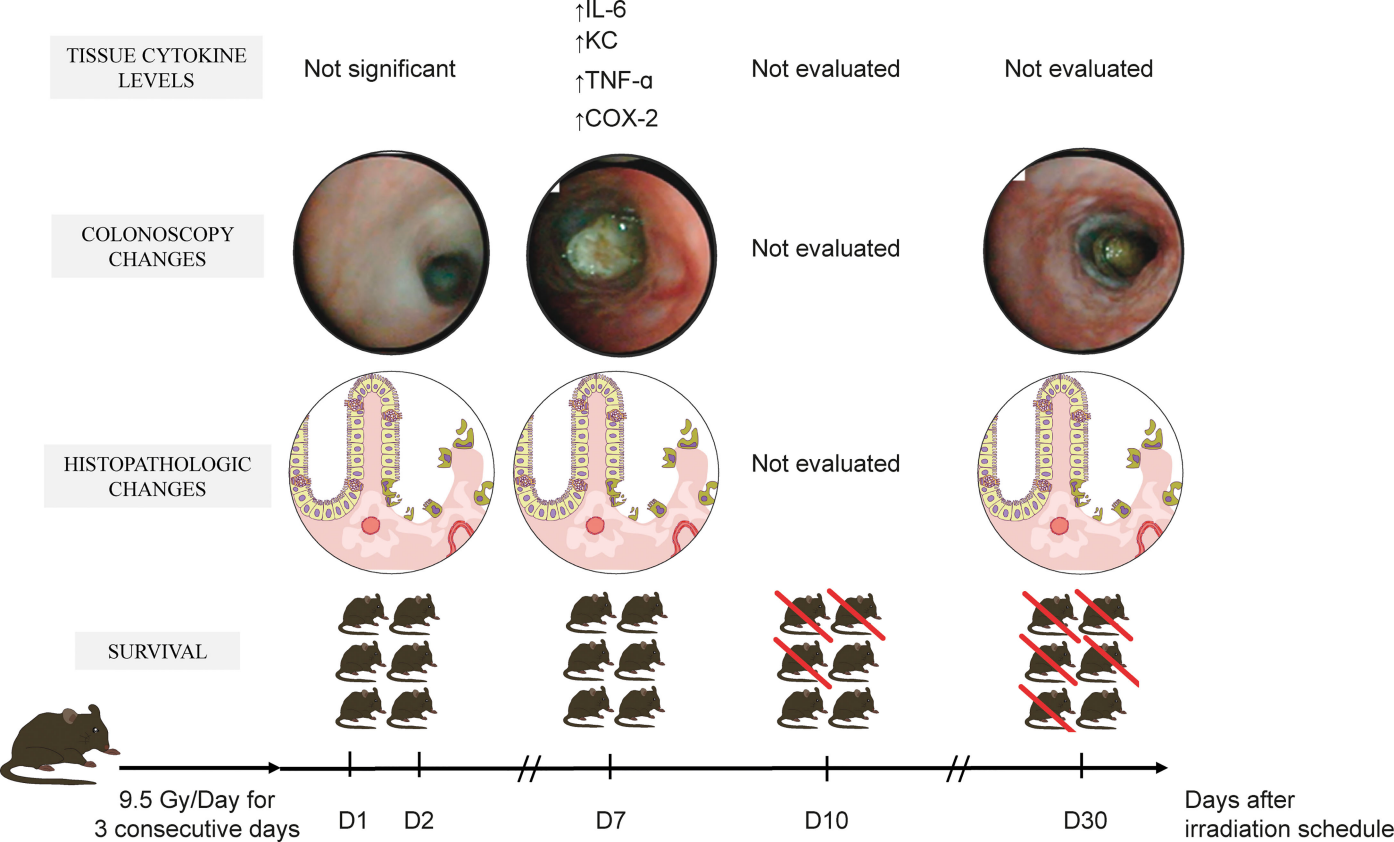
Schematic experimental protocol. The mice received a sham applicator into the rectum or were exposed to a high-dose-rate radiation source consisting of fractions of 9.5 Gy once a day for three consecutive days. The animals’ survival was then accompanied during 30 days, and the alterations at colonoscopy were determined on days 1, 2, 7, and 30. In another experimental setting, distal colon samples were obtained for histopathology. The optimal experimental day was chosen to further analyze inflammatory mediators by ELISA and immunohistochemistry. This figure describes the time-course of morphologic and biomarkers changes after radiation exposure. On days one and two, the mucosal injury was detected by histopathology. On day seven, macroscopic mucosal lesions were observable by colonoscopy. It was accompanied by increased tissue levels of inflammatory cytokines and enzymes. Half of the animals were alive on the tenth experimental day, and pronounced mortality was evident at day 30 (> 80%).

The animals exposed to radiation were previously submitted to light anesthesia with an intraperitoneal injection of ketamine (50 mg/kg) and xylazine (2 mg/kg). After animal immobilization, a lidocaine-soaked cylindrical applicator (3.1 mm outer diameter) was introduced into the rectum until its tip reached 20 mm far from the anal verge. We used the high-dose rate brachytherapy system HDR Microselectron (Nucletron, Elekta Medical Systems LTDA, São Paulo, Brazil) with an Iridium 192 (192Ir) source and 2.5 mm spacing between sources for proctitis induction. Each animal was irradiated individually in a supine position. The animals were divided into irradiated and sham groups. The first one was exposed to radiation consisting of fractions of 9.5 Gy once a day for three consecutive days. The target irradiated tissue was set 3.0 mm far from the applicator’s surface. On the other hand, the sham control group received the same cylindrical endorectal applicator for the same period as the experimental group but with no activation of the radiation source. After the last irradiation exposure, the survival was accompanied by 30 days. We hastened the euthanasia of animals with signs of imminent death, including piloerection, reduced locomotion, inability to maintain an upright position, ataxia, tremor, and altered breath frequency. Other groups of sham and irradiated mice were examined by colonoscopy on days 1, 2, 7, and 30 to detect signs of visible tissue damage, and they were euthanized for histopathological analysis. We determined the optimal experimental timeframe for animals’ euthanasia to harvest the tissue samples for further analysis based on these parameters. The schematic experimental design is depicted in **Figure 1**.

### Colonoscopy Analysis

A high-resolution mouse video endoscope (Tele Pack Vet X Led, Strattner, Karl Storz Endoskope, Rio de Janeiro, Brazil) was used for monitoring proctitis induction. The instrument consists of a video control system, a light source, a video recorder, a HOPKINS Forward Oblique flexible endoscope 30° (diameter 1.9 mm and length 10 cm), and a protective sheath (10). A researcher blind to the treatments performed the colonoscopy preceded by the local instillation of a 37°C saline enema. We used the sum of a four parameter-based tissue injury score system (range 0–12) to measure the proctitis severity (11), as follows: perianal findings, 0 (no findings), +1 (diarrhea), +2 (blood), +3 (rectal prolapse); transparency of the intestinal wall, 0 (vessels of all visible sizes, no thickening), +1 (large and medium vessels visible), +2 (large vessels barely visible), +3 (no visible vessels, maximum thickening of the mucosa); bleeding, 0 (no bleeding), +1 (bleeding due to endoscope contact), +2 (mild spontaneous bleeding), +3 (intense spontaneous bleeding); inflammatory lesions, 0 (no lesions), +1 (mucosal edema), +2 (erosions), +3 (mucosal ulceration).

### Histopathology Analysis

We fixed the samples in 10% neutral buffered formalin, followed by specimens’ dehydration and paraffin embedding. Histological sections cut at 5 µm were obtained for the hematoxylin-eosin staining (H&E) and examined by light microscopy (magnification × 100). Radiation injury score (RIS, cumulative score range 0–12) was a composite of seven histopathological alterations (12): serosal thickening, mucosal ulceration, epithelial atypia, vascular sclerosis, intestinal wall fibrosis, lymphatic congestion, and cystic alterations. Each parameter was graded as +0 (no changes), +1 (mild), +2 (moderate), +3 (intense injury).

### IL-6 and CXCL1/KC Dosage

According to the manufacturer, the interleukin-6 (IL-6) and keratinocytes-derived chemokine (KC) concentration was determined in samples of the rectum using an enzyme-linked immunosorbent assay (ELISA) (Catalog # DY406 and DY453, DuoSet ELISA Development kit, R&D Systems, MN, USA). Briefly, rat anti-mouse IL-6 or KC capture antibody-coated microtiter plates were blocked with 1% bovine serum albumin solution for 1 h. The sample and standards were added at various dilutions in duplicate and incubated at 4 °C for 2 h at room temperature. After washing the plates, biotinylated goat anti-mouse IL-6 or rat anti-mouse KC detection antibody (diluted reagent buffer 1% BSA) was added. After incubation at room temperature for 2 h followed by a washing step, streptavidin-HRP (diluted 1:200, 100 μl/well) was added. A substrate solution comprised of 100 µL of a 1:1 mixture of H_2_O_2_ and tetramethylbenzidine) was added to the plate and incubated in the dark at room temperature for 20 min. The enzyme reaction was stopped with 2N H_2_SO_4_, and the absorbance was measured at 450 nm. The results are expressed as pg/g of tissue and reported as the mean ± S.E.M.

### TNF- α and COX-2 Expression

Sample cross-sections were deparaffinized and rehydrated with xylene and graded alcohols. After antigen retrieval, the endogenous peroxidase was blocked, and the sections were incubated with primary rabbit anti-TNF-α antibody (1:100 in bovine serum albumin [BSA], ABCAM) or anti-COX-2 antibody (1:200 in BSA, Santa Cruz Biotechnology, USA) followed by incubation with biotinylated goat anti-rabbit antibody (diluted 1:800 in BSA, Santa Cruz Biotechnology, USA). The slides were washed and incubated with the avidin-biotin-horseradish peroxidase conjugate (Strep ABC complex by Vectastain^®^ ABC Reagent and peroxidase substrate solution), according to the Vectastain protocol (Vector Laboratories, Inc., Burlingame, CA, United States). Immunostaining was visualized with the chromogen 3,3’-diaminobenzidine (DAB). The negative control sections were processed simultaneously. The slides were counterstained with Harry’s hematoxylin, dehydrated in graded alcohol series, cleared in xylene, and coverslipped. TNF-α and COX-2 expressions were blinded scored based on the intensity of the staining, as follows: no staining (0); weak staining (1); moderate staining (2); moderate–intense staining (3); intense staining (4), according to Lima-Júnior and colleagues (13).

### Data and Statistical Analysis

We expressed the data as the means ± standard error of the mean, except for the histopathological and colonoscopy scores, reported as the median values (range). After running a normality test, the animal data were analyzed using Student’s t-test or Mann-Whitney U-test, as appropriate. The Mantel-Cox log-rank test was used to assess differences between survival curves. The tests were considered statistically significant when the *P*-value was < 0.05. Graph Prism version 8 (San Diego, CA, United States) was used for analysis.

## Results

During the model standardization, groups of mice were irradiated with 3 x 7.5 Gy or 3 x 9.5 Gy dose schedules (data not shown). A summary of the main experimental findings is presented in the **Supplementary Data Table 1**, which indicates the animals’ exposure to low radiation doses develop no tissue injury. No signs of the lesion were observed when the target irradiated tissue was set 0.5 mm far from the applicator’s surface despite the dose used (**Supplementary Data Table 1**).

### High-Dose Radiation Exposure Reduces Animals’ Survival

**Figure 2:**
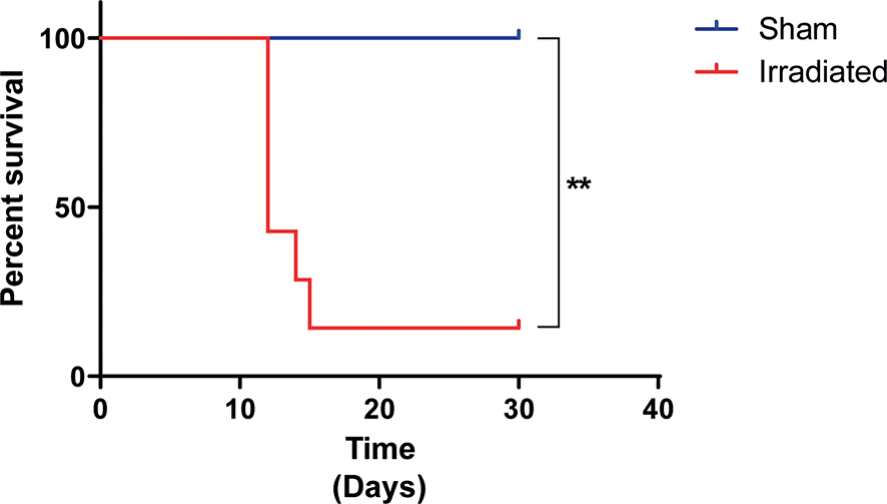
Irradiated mice presented reduced survival. The mice (n = 6 per group) were exposed to three fractions of 9.5 Gy or sham applicator once a day for three consecutive days. The animals’ survival was accompanied for 30 days. High-dose-rate radiation exposure reduces animals’ survival by 86%. **P < 0.05 vs. sham group.

HDR radiation exposure reduced the animals’ survival. As shown in **Figure 2**, the animals’ survival was accompanied for 30 days. Notably, more than 50% of mice died within ten days post three cycles of 9.5 Gy radiation exposure, contrasting with 100% survival of the animals that received the sham applicator (*P* < 0.01).

### Radiation Exposure Induces Intestinal Injury

We monitored by colonoscopy and histopathology the development of radiation-related tissue damage in the distal colon of animals at experimental days 1, 2, 7, and 30. The search for colonoscopy findings in the mice exposed to radiation indicated that the injury was significantly established (*P* < 0.05) in a time-dependent manner (**Table 1**) when the mice at the experimental day seven were compared with animals at earlier experimental time points. Additionally, the mice treated with radiation presented with moderate to intense intestinal alterations on the seventh day (colonoscopy score 9 [7-12], **Table 1**), which was statistically different (*P* < 0.05) from the sham group (0[0–0]). Visual inspection of the perianal region indicated diarrhea and phlogosis in the animals exposed to the radiation treatment.

**Table 1:** Colonoscopy and histopathologic scores.

Experimental day	Colonoscopy scores		Histopathologic scores	
	Sham Group	Irradiated Group	Sham Group	Irradiated Group
**1**	0 (0–1)	1 (0–5)	0 (0–1)	5 (1–9) *
**2**	0 (0–1)	0.5 (0–2)	0 (0–2)	4.5 (3–6) *
**7**	0 (0–0)	9 (7–12) *,#	1 (0–2)	11.5 (8–12) *,#
**30**	0 (0–2)	*n.d.*	0 (0–1)	*n.d.*

n.d. not determined. Data analysis from 30-day irradiated group impaired by the high animal mortality (not considered for the statistical analysis). *P < 0.05 vs the sham group; ^#^P < 0.05 vs the experimental day 2 of the irradiated group.

**Figure 3:**
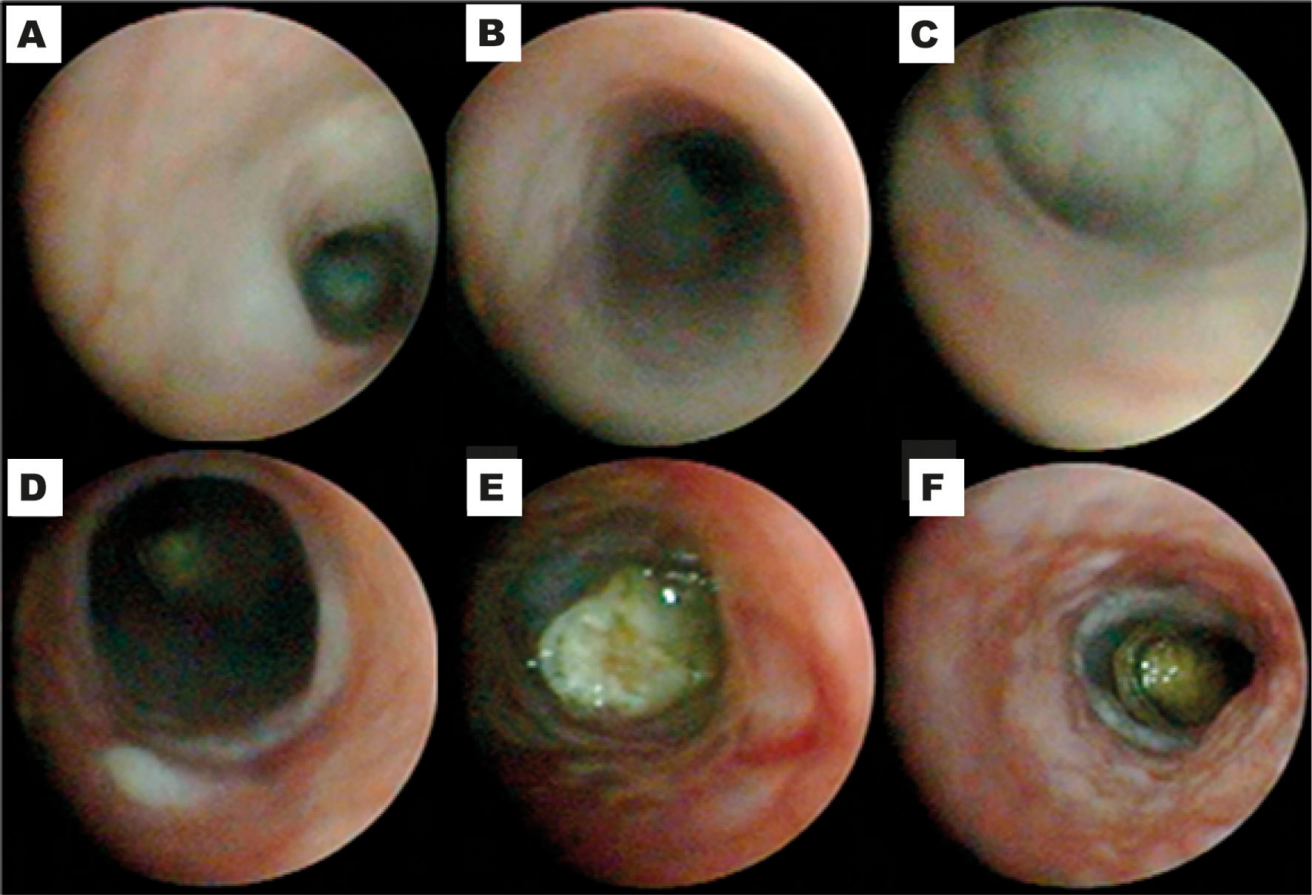
Irradiation-related tissue damage detected by colonoscopy. The animals received three fractions of 9.5 Gy or sham applicator once a day for three consecutive days. Tissue damage was assessed by colonoscopy on days 1, 2, 7, or 30. Standard tissue architecture is observed in the sham group **(A–C)**, while irradiated mice present with thickening of the mucosa **(D)**, moderate hemorrhagic areas **(E)**, and mucosal erosion **(D–F)**. The panels are representative of colonoscopy images obtained on experimental day seven.

**Figure 4:**
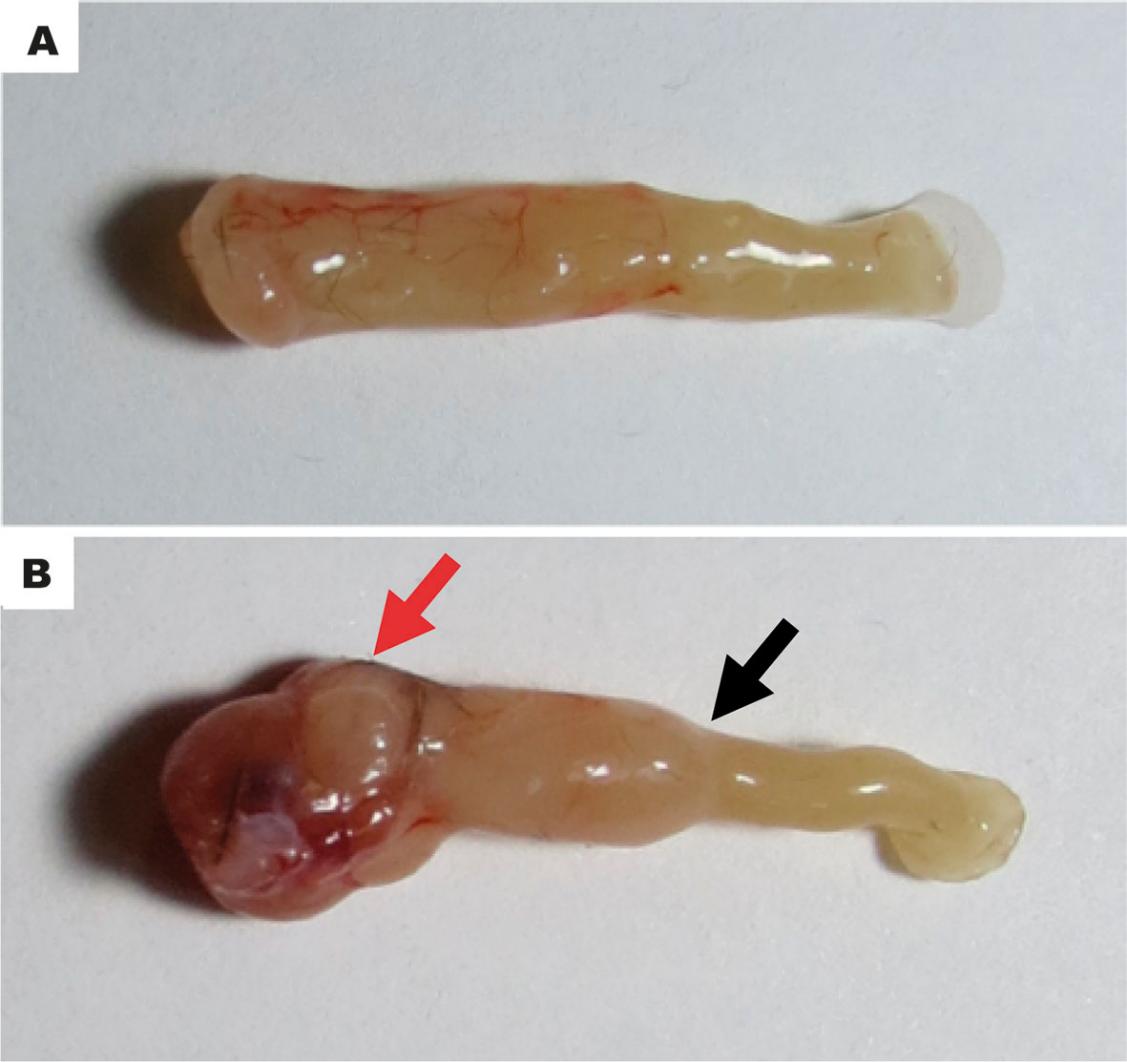
Representative macroscopy of tissue injury. The typical unaltered intestinal architecture of the sham group **(A)**. The intestinal sample obtained from irradiated mice shows wall thickness, hyperemia, hemorrhage, and inflammation **(B)**. The panels are representative of macroscopy images obtained on experimental day seven. Red arrow denotes a necrotic, hemorrhagic area. Black arrow indicates an area of stenosis.

As shown in **Figure 3**, the analysis by colonoscopy further evidenced large vessels barely visible and thickening of the mucosa (**Figure 3D**), accompanied by mild to intense spontaneous bleeding (**Figure 3E**) and inflammatory lesions marked by mucosal erosions and ulceration (**Figures 3D–F**). These findings diverged from the standard tissue architecture observed in the sham group (**Figures 3A–C**). The high mortality among the radiation-exposed animals (**Figure 2**) hampered the colonoscopy analysis at day 30 (**Table 1**). Representative macroscopy of the intestines harvested from the sham and irradiated mice is depicted in **Figures 4A, B**, respectively. **Figure 4B** depicts an intestine presenting wall thickness, hyperemia, hemorrhage, and inflammation, which contrasts with the normal tissue from the sham group (**Figure 4A**).

**Figure 5:**
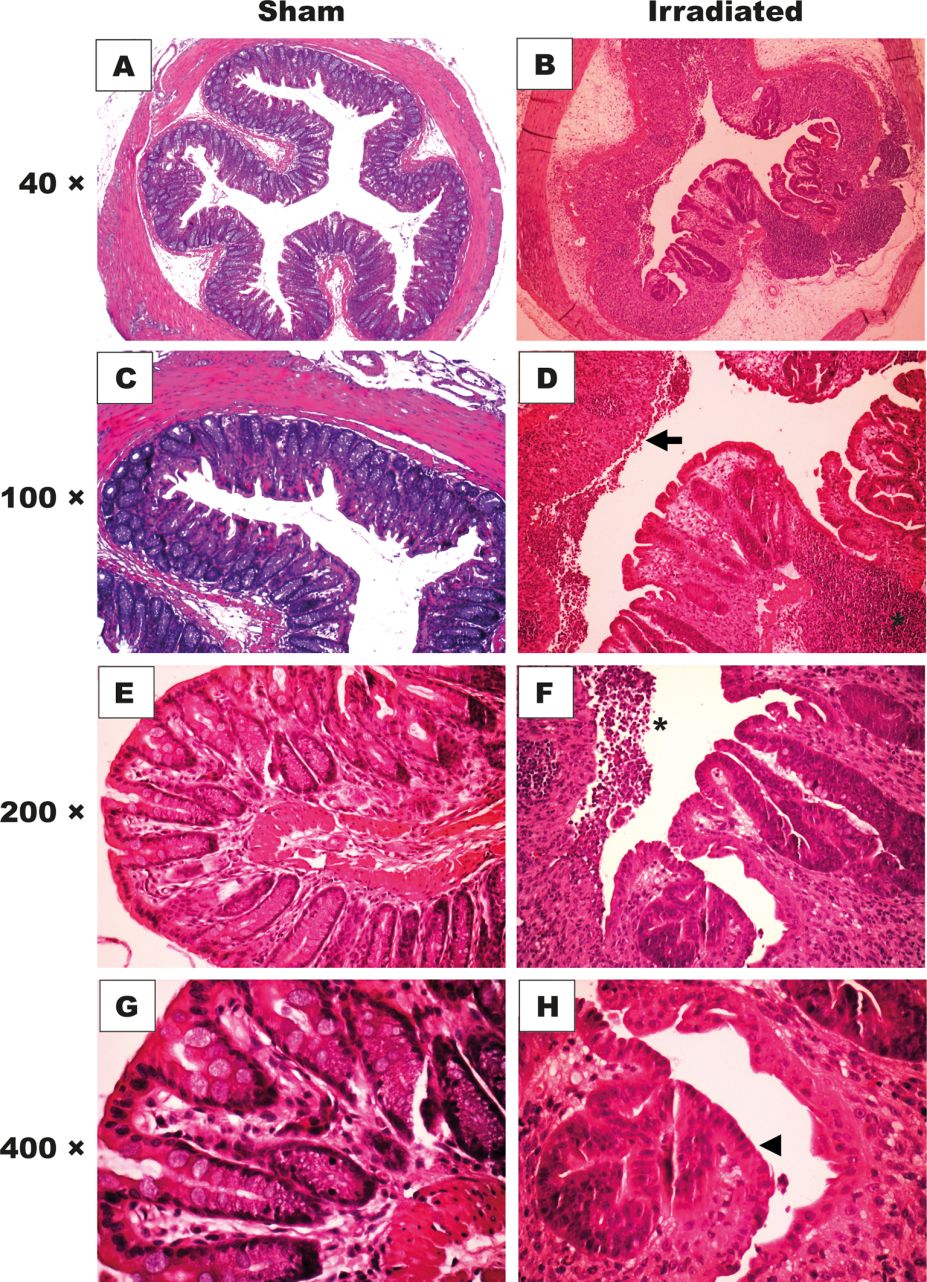
High-dose-rate brachytherapy induces histopathological alterations. C57BL/6 mice received three fractions of 9.5 Gy (n = 6) or sham applicator (n = 5) once a day for three consecutive days. For histopathological analysis, distal intestinal samples were obtained on days 1, 2, 7, and 30. H&E staining (× 40-400 magnification). The intestinal mucosa of the sham group shows preserved crypts and standard glandular architecture **(A, C, E, G)**. The mucosa of irradiated animals presents crypt disarrangement, and edema in the intestinal wall **(B)**, epithelial cell erosions (**D**, *arrow*), inflammatory infiltrate **(D, F)**, *asterisk*), and epithelial atypia (**H**, *head of an arrow*). The panels are representative of histopathology images obtained on experimental day seven.

After animals’ euthanasia on day seven, we collected distal intestinal samples for histopathology. The semi-quantitative analysis of the histopathological alterations (**Table 1**) indicated that the radiation exposure induced a time-dependent tissue injury, which was most pronounced at day 7 (11.5[8–12], *P* < 0.05 vs. days 1 and 2), contrasting with the typical findings of the sham group (1[0–2], *P* < 0.05). Histopathological damage was characterized by edema in the submucosa (**Figure 5B**), loss of glandular structures and the presence of mucosal ulceration (**Figure 5D**), and regenerative glandular epithelium (**Figure 5F**), vascular stenosis, lymphatic vessel dilation, and serosa thickening. Glandular epithelial regeneration with atypia was also observable (**Figure 5H**). These findings contrast with the unaltered histopathological architecture of samples from the sham group (**Figures 5A, C, E, G****)**.

### Inflammatory Markers Increase After Irradiation

**Figure 6:**
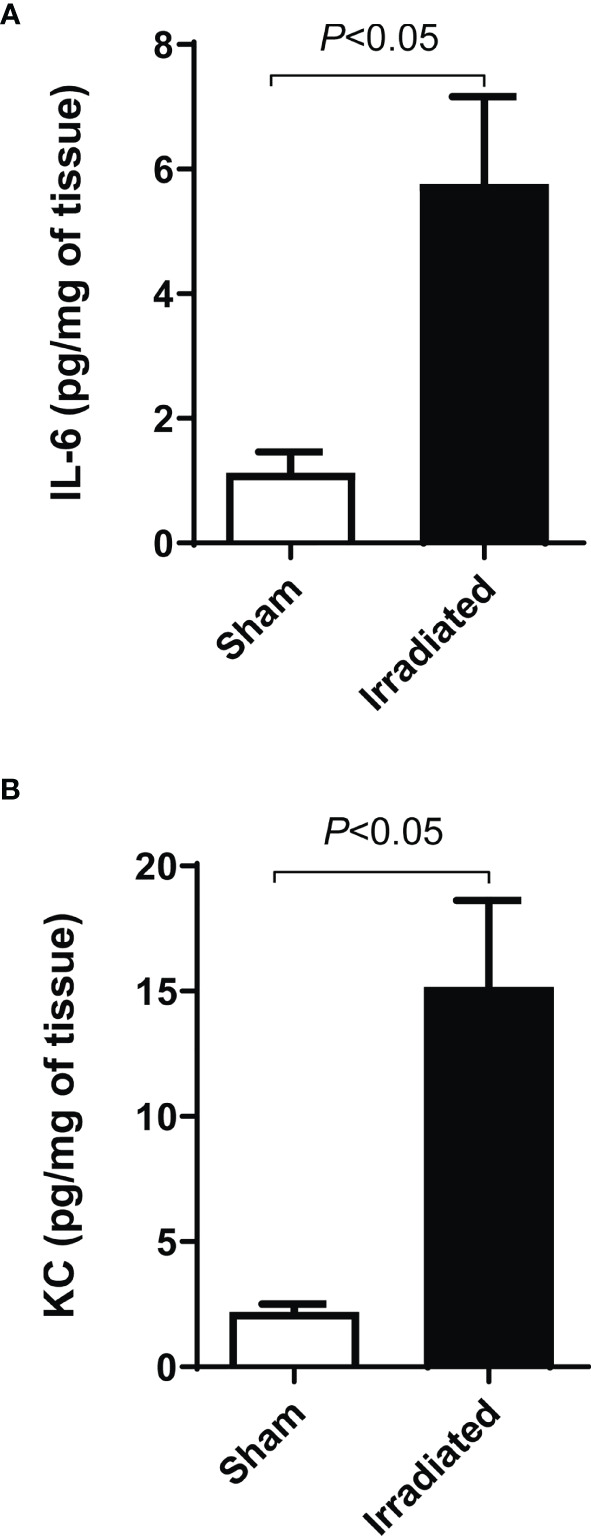
Colonic irradiation increases the tissue levels of inflammatory cytokines. The animals received a sham applicator into the rectum (n = 5) or were exposed to a high-dose-rate radiation source consisting of fractions of 9.5 Gy once a day for three consecutive days (n = 6). Intestinal samples were harvested for IL-6 and KC dosage by ELISA. Irradiated mice presented elevated levels of IL-6 **(A)** and KC **(B)** compared with the sham group. Data are expressed as the mean ± SEM and were analyzed by the Student’s t-test.

**Figure 7:**
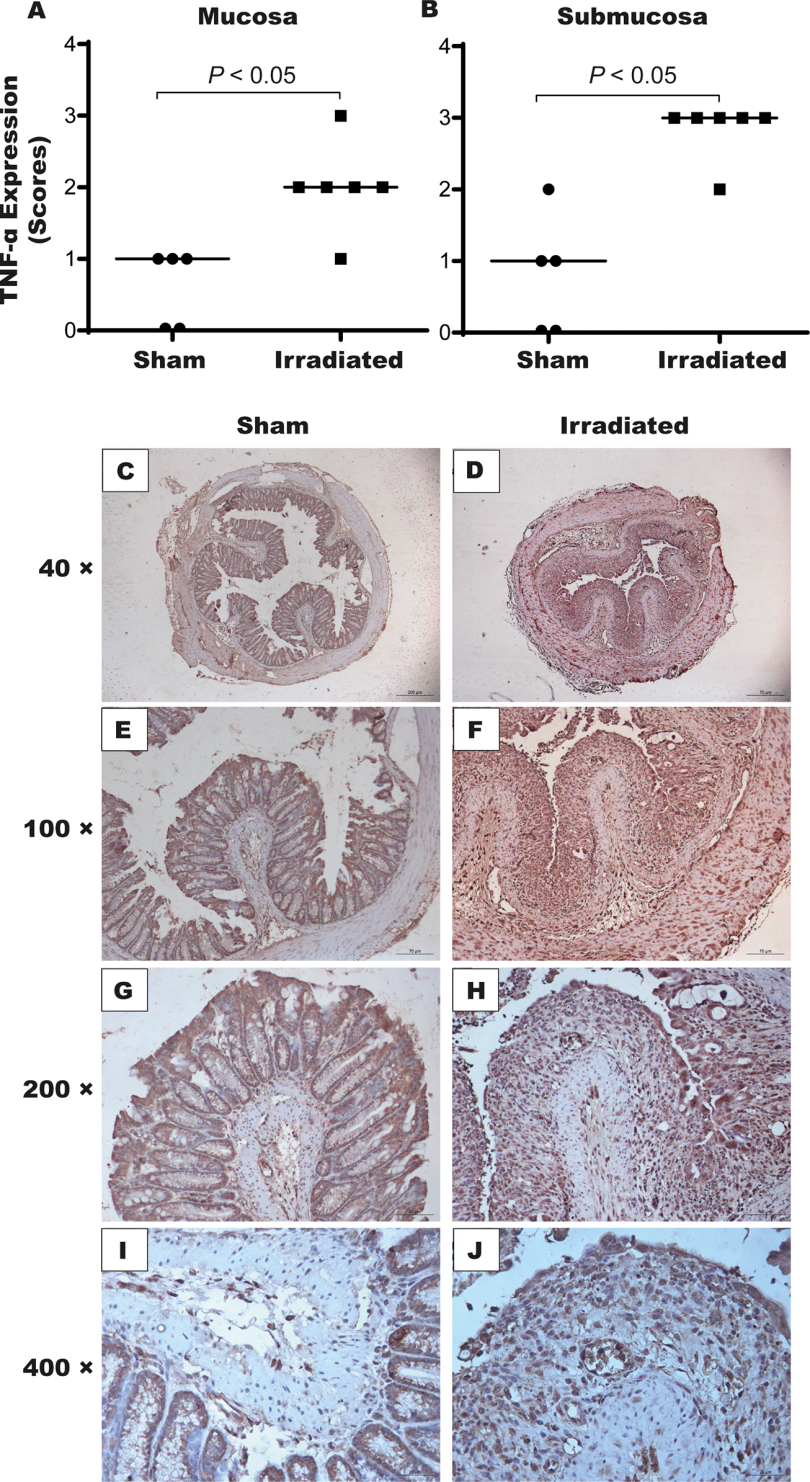
High-dose-rate brachytherapy augments TNF-α expression in the intestinal tissue of mice. For the immunohistochemical assay, sham and irradiated animals (n = 5–6 per group) were euthanized on day seven. Semi-quantitative analysis indicated increased TNF-α expression in the mucosa **(A)** and submucosa **(B)**. Panels **(C–J)** depicts the stained cells, which are more intense in the irradiated group. Data were analyzed by the Mann–Whitney’s test. *P* < 0.05 indicates the statistical difference between the groups. (× 40-400 magnification).

**Figure 8:**
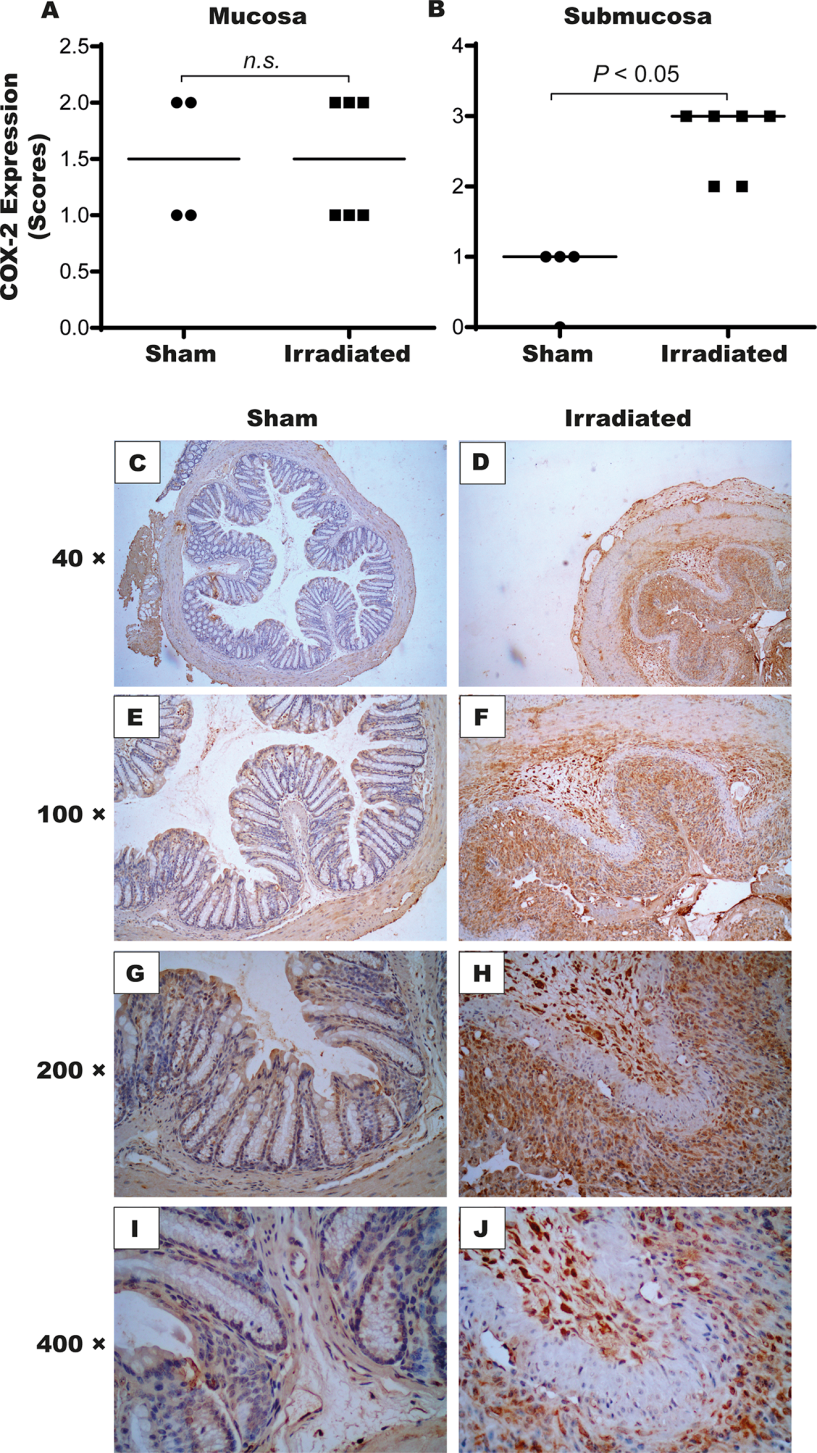
High-dose-rate brachytherapy increases COX-2 expression in the intestinal tissue of mice. For the immunohistochemical assay, sham and irradiated animals (n = 5–6 per group) were euthanized on day seven. Semi-quantitative analysis indicated increased COX-2 expression in the submucosa **(B)** but not in the mucosa **(A)**. Panels **(C–J)** depicts the stained cells, which are more intense in the irradiated group. Data were analyzed by the Mann–Whitney’s test. *P* < 0.05 indicates the statistical difference between the groups. (× 40-400 magnification). ns, denotes not significant.

The inflammatory process markedly manifested seven days post-radiation exposure. Notably, IL-6 (**Figure 6A**) and KC (**Figure 6B**) levels respectively increased 590% and 690% in the distal intestinal tissues obtained from irradiated mice compared with the sham group (*P* < 0.05). No statistical difference was detected between the groups on the experimental days one and two (**Supplementary Data Figure 1**). Additionally, the immunohistochemical analysis revealed that TNF-α expression augmented in the mucosa (2[1–3], **Figure 7A**) and submucosa (3[2–3], **Figure 7B**) of the irradiated group versus the sham group (mucosa: 1[0–1], and submucosa 1[0–2], *P* < 0.05). However, COX-2 expression was found to increase in the submucosa (3[2–3], **Figure 8B**) but not in the mucosa (1.5[1–2], **Figure 8A**) of mice exposed to radiation. Representative expression of TNF-α and COX-2 is depicted in **Figures 7C–J** and **8C–J**, respectively.

## Discussion

The present study designed an experimental model of HDR actinic proctitis characterized by colonoscopy and histopathological changes and pro-inflammatory mediator production. The time course indicated that the constellation of changes ideally manifested on day seven post-radiation exposure with no loss of mice at that time point. Long-term analyses were not viable given the high animal mortality.

Actinic proctitis is considered a significant downside of radiotherapy in oncology. Radiation-induced severe rectal damage might be accompanied by fistulae and hemorrhage, contributing to poor therapeutic outcomes. Despite the improvement in radiation therapy techniques, the management of this pathological condition remains a challenge in the clinical setting (14). Currently, there are no available guidelines or good-evidenced-based therapy to prevent or treat this condition, but several therapeutic measures have been proposed with variable results (1). Selective targeting of specific driving inflammatory mediators might then be a promising strategy.

Animal models that closely mimic the clinical condition are essential to understanding the underlying pathophysiology. Currently, only two rodent models have been proposed (7, 8). Symon and colleagues developed an HDR brachytherapy-based proctitis mouse model in which the animals were exposed to radiation dose schedules of 3 x 5.5 (biologically effective dose (BED) of 39.2 Gy4) to 5 x 8 Gy (BED of 94 Gy4). However, the tissue damage assessments were restricted to the chronic phase of the disease (8). Those authors demonstrated a positive correlation between inflammatory cytokines, such as IL-1β and IL-6, and histologic scores (8). Another elegant study used an X-RAD 225-Cx (Precision X-Ray) small animal irradiator, allowing multiple planning configurations of treatment volume and organ-at-risk avoidance. The authors delivered a single 15 Gy 3D (BED of 71.2 Gy4) conformal treatment plan and accompanied the animals for 250 days (7). Interestingly, most analyses were performed more than ten weeks after the animal irradiation procedure (7). Despite the convincing conclusions obtained in those studies, more feasible animal models are needed to overcome methodological difficulties that limit the reproducibility of clinical disease. Remarkably, irradiated cancer patients that experience acute actinic proctitis are about five times more likely to develop late manifestations than those who are asymptomatic (15). Although such association remains controversial (16), implementing a pharmacological modulation of the acute condition might be a window of opportunity.

The three dose fractions of 9.5 Gy (BED = 96.2 Gy4) used in the present study allowed the development of a complete spectrum of clinical symptoms in only one-week post-radiation exposure (7, 8). Remarkably, the dose of 3 x 9.5 Gy used in the experiments is equivalent to 64 Gy in conventional fractionation of 2 Gy per daily fraction considering an alpha/beta ratio of 4 in the clinical setting, which is below the acceptable limit for small volumes of the irradiated rectum.

First, we tested the optimal conditions for tissue irradiation to induce the inflammatory response in the shortest timeframe as possible. The applicator used in our study considered the largest diameter (3.1 mm) possible to prevent discomfort and suffering to the animals even under anesthesia. Additionally, mechanical injury associated with large applicators could be a confounding factor during the analysis. Prescription of 3 x 9.5 Gy at the depth specified in our study (3 mm from the applicator’s surface) meant a dose of 3 x 21 Gy on the surface, which may explain the high mortality observed after ten days in the irradiated group. However, the same dose (3 x 9.5 Gy) prescribed at a depth close to the applicator surface (0.5 mm, the smallest prescription depth possible by our planning system) was ineffective in causing the expected damage (data not shown). Then, we proceeded with the 3 x 9.5 Gy tissue irradiation applied 3.0 far from the applicator’s surface. The manifestation of an insidious lesion in this model markedly reproduced the acute and chronic clinical findings with the presence of telangiectasia, vascular sclerosis, inflammation, and tissue necrosis (3). The inflammatory response is a condition commonly associated with pain and distress, which negatively affects the animal’s well-being (17). As demonstrated in our study, the high dose of brachytherapy-induced actinic proctitis induces a high mortality rate ten days post-radiation exposure, which is associated with an inflammatory lesion in the colon. The animals’ survival study was essential to provide vital information for designing the model. Subsequent experiments were conducted by euthanizing the mice on the experimental day seven since the inflammation was well-established in the colon, as detected by colonoscopy and confirmed by histopathology and expression of inflammatory markers. Notably, the intense characteristics of the disease impaired longer animal follow-up. The colonoscopy changes were then considered the primary endpoint of this study since it macroscopically characterized the lesion was established. Such a decision also considered the preliminary experimental findings in the pilot study, in which we assessed the time-course of IL-6 and KC production, presenting statistically significant only at day seven.

The high turnover activity of the gastrointestinal tract contributes to its radiosensitivity (18). Ionizing radiation activates oxygen free radicals, injures the DNA, and disorganizes cellular structures, compromising cell function (19). Interestingly, the presence of mucosal ulceration and loss of glandular structures seen at histopathology corroborate the direct mechanism of injury caused by the radiation. As confirmed by colonoscopy, the altered blood supply to the intestinal wall possibly leads to intestinal ischemia following radiation, potentiating the damage (20). The injured rectum architecture exposes the lamina propria to luminal bacteria activating an inflammatory response (21), involving T-lymphocytes, macrophages, and neutrophils (20).

The activation of the inflammatory process in our model is likely triggered by pathogen-associated molecular patterns from lumen bacteria and damage-associated molecular patterns from dying cells. Homeostasis breakdown activates an organized and hierarchical production of mediators, mainly TNF-α and IL-1 family of cytokines followed by chemokines, COX-2, and lipid mediators (22). These mediators orchestrate vascular changes and immune cell influx into the injured tissue. Particularly, we found increased levels of TNF-α, IL-6, KC, and COX-2 in tissue samples of the irradiated group, indicating the involvement of these mediators in the pathogenesis of actinic proctitis. These findings are in line with another study in which the expression of toll-like receptors, matrix metalloproteinases, chemokines, and inflammatory enzymes augmented in the rectum of pigs exposed to high radiation dose to induce actinic proctitis (14). Identifying crucial inflammatory markers opens the perspective on their target modulation in pathological conditions. For instance, autologous bone marrow-derived mesenchymal stem cells injection switches the microenvironment from pro-inflammatory toward an anti-inflammatory response, preventing tissue damage and fibrosis in a pig model of proctitis (14), probably by inducing an immunosuppressive environment (23). There are currently several drugs prescribed for inflammatory conditions that could apply for actinic proctitis clinical management, including glucocorticoids, COX-2 inhibitors and monoclonal antibodies, such as infliximab. The mediators identified in this study are the first potential targets to be explored. As mentioned above, the severity of this side-effect might involve pathogenic intestinal bacteria, whose modulation could also be considered.

The development of animal models that mimic human diseases requires some essential adaptations. The main one includes the need for about 100% of animals to express the disease to evaluate potential pharmacological therapies. Such requisite considers the reduced number of animals per experimental group. On the other hand, variable disease manifestation in which not all animals manifest the disease implies much larger testing groups. That would closely represent the clinical setting. However, the ethics committees constantly demand researchers to refine the experimental methods, reduce the number of animals per group, and replace animals by computer models or invertebrates. Therefore, animal models are commonly designed by submitting them to more intense dose regimens to establish the constellation of symptoms in the shortest term as possible in all individuals exposed. Such aggressive disease-causing high animal mortality in the mid- and long-term could partially explain the difficulty of translating basic research findings to the bench. It is a potential limitation of this study.

In conclusion, we proposed a novel and feasible animal model of actinic proctitis that in one week reproduces acute and chronic findings commonly manifested in the rectum of patients treated with HDR brachytherapy. The advantage of using mice in this model involves their small size, ease of maintenance, and the effective and efficient reproducibility of human diseases (24), overcoming the housing limitations of larger animals used in other studies. The inflammatory mediators identified in this study might open the way to future clinical applications as pharmacological targets. The inhibition of the acute inflammatory response could reduce the emergence of limiting chronic symptoms that patients experience more than six months post radiation exposure. Less aggressive dose regimens different from the one chosen in this research are highly welcome as an alternative to evaluate long-term effects of tissue irradiation, provided an acceptable animal’s survival is observable.

## Data Availability Statement

The original contributions presented in the study are included in the article/**Supplementary Material**. Further inquiries can be directed to the corresponding authors.

## Ethics Statement

The animal study was reviewed and approved by Ethics Committee on Animal Use from the Federal University of Ceará, approval number 50/13. This study complied with the ARRIVE Guidelines 2.0 (25).

## Author Contributions

CHBL, FC, and RL-J were responsible for the design and implementation of the project. CHBL, CDHL, CAVGL, DT, GL, and JF were responsible for the data acquisition. CHBL, CDHL, PA, DW, and RL-J were in charge of data statistics and analysis. PA was in charge of histopathological specimen analysis. CHBL was responsible for irradiation procedures. FC and RL-J were responsible for funding acquisition. CHBL, CDHL, DW, and RL-J wrote the paper. All authors contributed to the article and approved the submitted version.

## Funding

This work was supported by CNPq (Conselho Nacional de Desenvolvimento Científico e Tecnológico, grant number: 421202/2018-1 and 310568/2017-0), CAPES (Coordenação de Aperfeiçoamento de Pessoal de Nível Superior, Finance Code 001, grant number: CAPES-PROEX 0756/2020), and FUNCAP (Fundação Cearense de Apoio ao Desenvolvimento Científico, grant number: PR2-0101-00054.01.00/15). Open access publication fee was funded by PRONEX/FUNCAP/CNPQ, Grant number PR2-0101-00054.01.00/15.

## Conflict of Interest

The authors declare that the research was conducted in the absence of any commercial or financial relationships that could be construed as a potential conflict of interest.

The reviewer GV declared a shared affiliation, with one of the authors FC to the handling editor at the time of the review.

The handling Editor declared a past collaboration with one of the authors RL-J.

## Publisher’s Note

All claims expressed in this article are solely those of the authors and do not necessarily represent those of their affiliated organizations, or those of the publisher, the editors and the reviewers. Any product that may be evaluated in this article, or claim that may be made by its manufacturer, is not guaranteed or endorsed by the publisher.
