# Supplementary material for: A Novel Murine Model of a High Dose Brachytherapy-Induced Actinic Proctitis
Source: Front Oncol. 2022 Feb 23;12:802621. doi: 10.3389/fonc.2022.802621 (PMC8909144; doi:10.3389/fonc.2022.802621)
Supplement: Supplementary file 1 [file DataSheet_1.pdf]

Supplementary Materials

A Novel Murine Model of a High Dose Brachytherapy-Induced Actinic Proctitis

Carlos Heli Bezerra Leite[1,*], Carlos Diego Holanda Lopes[2], Caio Abner Vitorino Gonçalves Leite[2], Dulce Andrade Terceiro[2], Gabriel Silva Lima[2], Jéssica Andrade Freitas[2], Fernando Queiroz Cunha[3], Paulo Roberto Carvalho Almeida[4], Deysi Viviana Tenazoa Wong[2], Roberto César Pereira Lima-Junior[2,*]

[1]*Radiation Oncology Service, Haroldo Juaçaba Hospital, Cancer Institute of Ceará (ICC), Brazil*

[2]*Drug Research and Development Center, Department of Physiology and Pharmacology, Faculty of Medicine, Federal University of Ceara, Brazil*

[3]*Department of Pharmacology, School of Medicine of Ribeirão Preto, University of São Paulo, Brazil*

[4]*Graduate Program in Pathology, Department of Pathology and Forensic Medicine, Faculty of Medicine, Federal University of Ceara, Brazil*

*** Correspondence:**
Carlos Heli Bezerra
chbl.13@hotmail.com

Roberto César Pereira Lima-Júnior
robertocesar@ufc.br

Supplementary Figure 1

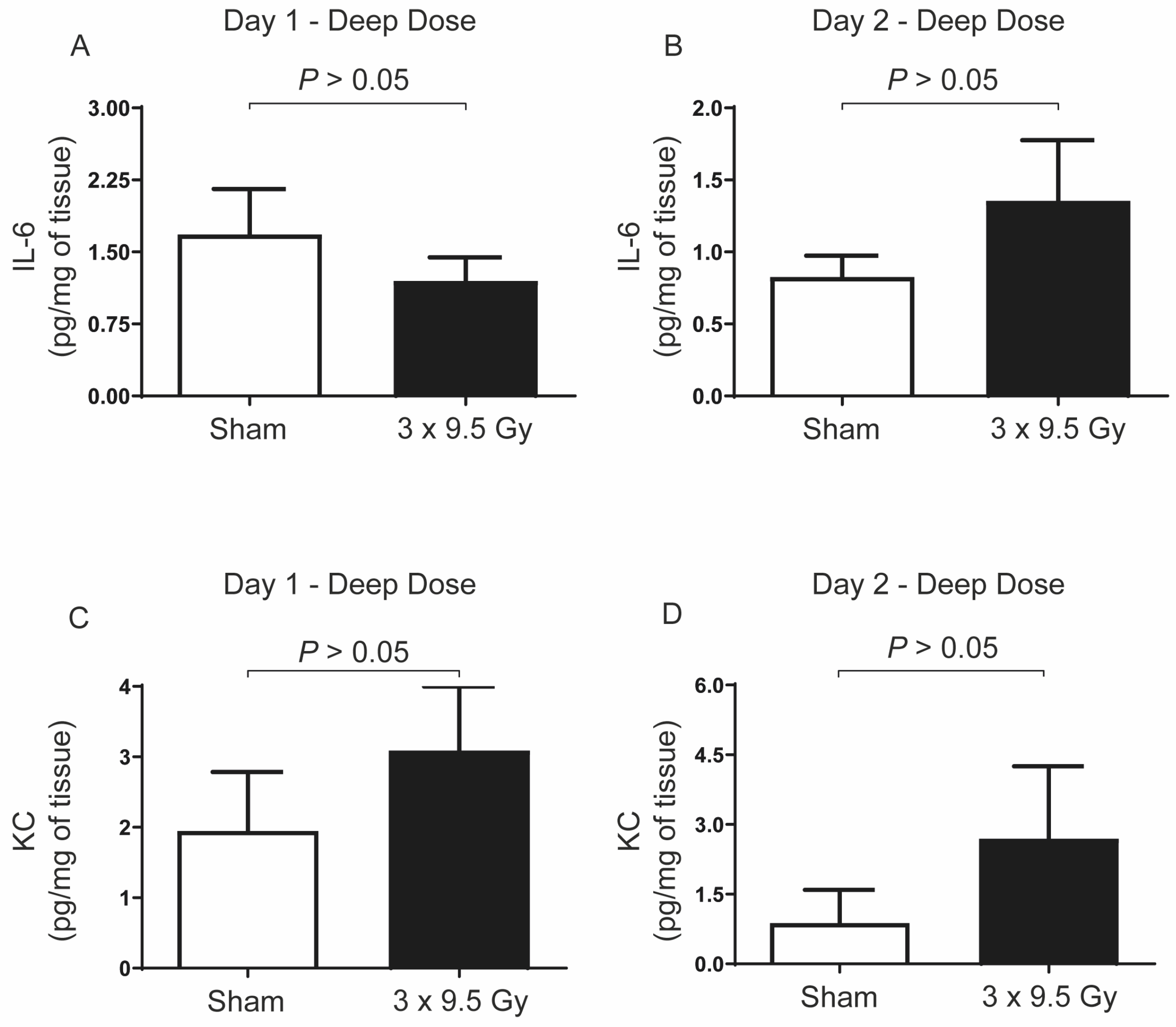

Colonic irradiation does not change tissue levels of inflammatory cytokines at experimental days 1 and 2. The animals received a sham applicator into the rectum (n=5) or were exposed to a high-dose-rate radiation source consisting of fractions of 9.5 Gy once a day for three consecutive days (n=6), 3 mm far from the applicator's surface. Intestinal samples were harvested for IL-6 (A and B) and KC (C and D) dosage by ELISA. There is no statistical difference between the groups. Data are expressed as the mean ± SEM and were analyzed by the Student's t-test.

Supplementary data table 1. Summary of the main experimental findings.

Depth of irradiation dose	Superficial Dose (0.5 mm far from the applicator's surface)							Deep Dose (3.0 mm far from the applicator's surface)			
**Time (Days)**	**1**		**2**		**7**	**30**		**1**	**2**	**7**	**30**
**Number of mice per irradiated group******	**5**	**5**	**5**	**5**	**5**	**5**	**5**	**6**	**6**	**6**	**6**
**Dose Intensity**	**3 x 7.5 Gy**	**3 x 9.5 Gy**	**3 x 7.5 Gy**	**3 x 9.5 Gy**	**3 x 9.5 Gy**	**3 x 7.5 Gy**	**3 x 9.5 Gy**	**3 x 9.5 Gy**	**3 x 9.5 Gy**	**3 x 9.5 Gy**	**3 x 9.5 Gy**
**Loss of Body Mass**	No	No	No	No	No	No	No	No	No	No	Yes
**Reduced Survival**	No	No	No	No	No	No	No	No	No	No	Yes
**Perineal Lesion**	No	No	No	No	Yes	Yes	Yes	No	No	Yes	*not tested****
**Colonoscopy changes**	No	No	No	Yes*	Yes*	Yes*	Yes*	No	No	Yes**	*not tested****
**Histopathologic changes**	No	No	No	No	Yes	Yes	No	Yes	Yes	Yes	*not tested****
**Increased tissue levels of KC**	*not tested*	*not tested*	*not tested*	*not tested*	*not tested*	No	No	No	No	Yes	*not tested****
**Increased tissue levels of IL-6**	*not tested*	*not tested*	*not tested*	*not tested*	*not tested*	No	No	No	No	Yes	*not tested****
**IHC for TNF-alpha**	*not tested*	*not tested*	*not tested*	*not tested*	*not tested*	*not tested*	*not tested*	*not tested*	*not tested*	Yes	*not tested****
**IHC for COX-2**	*not tested*	*not tested*	*not tested*	*not tested*	*not tested*	*not tested*	*not tested*	*not tested*	*not tested*	Yes	*not tested****

*Mild changes; **Intense changes; ***Impossibity to run the analysis due to the high animal mortality; **** The sham groups included five mice per experiment.
